# The incidence of regression after the non-surgical treatment of symptomatic lumbar disc herniation: a systematic review and meta-analysis

**DOI:** 10.1186/s12891-020-03548-z

**Published:** 2020-08-10

**Authors:** Yi Wang, Guogang Dai, Ling Jiang, Shichuan Liao

**Affiliations:** 1 Cervicodynia/Omalgia/Lumbago/Sciatica Department 2, Sichuan Provincial Orthopedics Hospital, 132 West First Section First Ring Road, Chengdu, 610041 Sichuan Province China; 2 College Hospital, Sichuan Agricultural University-Chengdu Campus, 211 Huimin Road, Wenjiang District, Cheng Du, Sichuan Province China

**Keywords:** Lumbar disc herniation, Non-surgical treatment, Incidence of regression

## Abstract

**Background:**

Although the regression of symptomatic lumbar disc herniation (SLDH) has been widely reported, little data exist regarding the generalized incidence of regression (IR). We aimed to review the varying IRs and to synthesize the pooled IR of non-surgically-treated SLDH.

**Methods:**

Four electronic databases were searched for relevant studies pertaining to the regression of SLDH after non-surgical treatment and for potential studies that may have reported morphological changes in lumbar disc herniation in the follow-up results of SLDH patients treated non-surgically. The main outcome was the regression of SLDH. A random effects model was used to determine the pooled IR of SLDH.

**Results:**

We identified 13,672 articles, 38 of which were eligible for analysis. Our analysis included 2219 non-surgically treated SLDH patients, 1425 of whom presented regression. The pooled IR was 63% (95% CI 0.49–0.77). In subgroup analyses, studies that quantitatively measured the regression of SLDH yielded statistically higher pooled IRs than those that used qualitative methods. The pooled IRs gradually increased in randomized controlled trials and prospective and retrospective studies. The pooled IR varied from 62 to 66% after the sequential omission of any single study. Meta-regression showed that study types, herniation levels and regression measurements caused heterogeneity.

**Conclusions:**

We report an overall IR of 63% among non-surgically treated SLDH patients, thus providing clinical decision makers with quantitative evidence of IR. Based on our systematic review, we suggest a follow-up timeline with time points 4 and 10.5 months after onset when deciding whether to perform surgery for SLDH.

## Background

Symptomatic lumbar disc herniation (SLDH) can be treated non-surgically or surgically. Non-surgical treatment was shown to be effective for SLDH long ago [1], although surgery results in more rapid and effective short-term alleviation of symptoms than non-surgical treatment [2, 3]. However, the long-term effects of the two have not been consistently reported [2–4], and there is a risk of complications with surgery [5]. Thus, in many cases, there is not a clear correct decision regarding the use of surgical or non-surgical treatments for SLDH [6].

Since the first case of regression after the non-surgical treatment of SLDH was reported in 1984 [7], the phenomenon of SLDH regression has been widely reported [8–46], with the incidence of regression (IR) varying from study to study. Reports on the correlation between the regression of SLDH and clinical outcomes have been contradictory: an early study observed a connection between morphological changes in SLDH and clinical outcomes [41], while later studies found that the regression of SLDH does not correspond with the resolution of symptoms [9, 47]. However, we cannot ignore the physical decompression that occurs during regression in the acute context of SLDH, and the probable regression of SLDH still needs to be considered in clinical practice, according to the guidelines of the North American Spine Society [48]. Understanding the IR of SLDH is clearly of clinical importance. However, scant generalized data regarding the IR are currently available to serve as a reference. When making clinical decisions regarding SLDH, practitioners and patients have little high-level evidence regarding IR to which they can refer.

We therefore performed a systematic review and meta-analysis to provide a comprehensive examination of the IR of SLDH in patients who were treated non-surgically.

## Methods

This systematic review and meta-analysis is reported in compliance with the Preferred Reporting Items for Systematic Reviews and Meta-Analyses (PRISMA) statement [49]. We did not publish a prior protocol for this systematic review and meta-analysis.

### Search strategy

For this systematic review and meta-analysis, we searched PubMed, Embase, the Cochrane Central Register of Controlled Trials, and the Web of Science (from inception to September 16, 2019). Search terms included those related to intervertebral disc herniation, regression, comparison, outcome, follow-up, image, and their variants. To avoid missing articles without information about the language in the database records, there was no language limitation in the literature search. A sample search strategy can be found in an additional file. We included studies identified from the references of included articles and other review articles on the topic. Two reviewers performed the searches. Disagreements were resolved by discussion with a third reviewer.

### Eligibility and exclusion criteria

Relevant articles pertaining to the phenomenon of the regression of SLDH after non-surgical treatment and potential studies that may have reported morphological changes in lumbar disc herniation (LDH) among the follow-up results for non-surgically-treated SLDH patients were included, with the publication language restricted to English. Randomized controlled trials (RCTs) and nonrandomized studies were eligible for inclusion. The following studies were excluded: 1. Studies that only reported the follow-up results of surgery, including percutaneous endoscopic transforaminal discectomy, microendoscopic discectomy, microdiscectomy, fenestration discectomy, open discectomy, lumbar laminectomy, lumbar interbody fusion and radiofrequency ablation; 2. Studies on cervical discs; 3. Studies that did not report the morphological changes in SLDH; 4. Studies that did not report the number of patients exhibiting regression; 5. Studies on only intradiscal injections, including oxygen-ozone therapy, plasma injection and collagenase chemonucleolysis; 6. Studies on asymptomatic LDH; 7. Studies with less than 10 patients at follow-up; 8. Animal studies; 9. Reviews; and 10. Studies that did not report specific non-surgical treatment.

### Quality assessment

The quality of the nonrandomized studies was assessed based on the Methodological Index for Nonrandomized Studies (MINORS) [50]. There is no consensus on when the regression of LDH occurs; thus, item six of the MINORS (follow-up period appropriate to the aim of the study) was not applicable, and the highest total score was 14 (high quality: 10–14; moderate quality: five-nine; and low quality: zero-four). The risk of bias of RCTs was evaluated using a tool from the Cochrane Collaboration [51]. Considering the nature of RCTs of the non-surgical treatment of SLDH, performance bias was generally not a particular concern and had a minor impact on the study quality. Thus, we considered all the included RCTs to have a low risk of performance bias. RCTs were categorized as having a high, low, or unclear risk according to the following criteria: high risk, any item presented a high risk; low risk, no more than 2 items presented an unclear risk; and unclear risk, more than 2 items presented an unclear risk. Two reviewers independently assessed the quality of the included studies and extracted the data. Disagreements were resolved by consensus with a third reviewer.

### Data extraction and analysis

Relevant data were extracted using a standardized form that included the publication year, country, study type, study quality or risk of bias, LDH level, regression measurement, imaging method, patient count, total number of SLDH patients at follow-up and number of patients with SLDH regression, as well as age, symptom duration, nerve symptoms, whether regression was defined and follow-up duration. The primary outcome was the IR of SLDH after non-surgical treatment. The IR was estimated based on the total number of SLDH patients at follow-up and the number of patients that experienced regression. For studies that recorded the number of patients according to the regressed proportion or size interval but did not define the interval of non-regression or the number of patients without regression, we regarded the lowest interval as the no-regression range, and the number of patients outside of this interval was considered the number of patients with regression. For studies in which more than two imaging examinations were performed, we used the author’s final count, and if no final count was provided, the latest imaging examinations with enough information were compared to the baseline examinations. For studies reporting the same cohort or trial, only the latest study was included. For studies with overlapping data, we selected the study with the highest number of patients at the last follow-up. Herniations after baseline were not counted. For RCTs, we calculated the total number of occurrences in the two groups.

The I^2^ statistic was employed to evaluate the heterogeneity of pooled data, and the DerSimonian and Laird random effects model was used to pool the IRs with corresponding 95% confidence intervals (CIs). Incidences from studies with zero events were treated by adding 0.5 cases to both the numerator (number of patients with regression) and denominator (total number of SLDH patients), consistent with recommended practices [52]. Subgroup analysis was performed by stratifying the studies according to the time period, region, study type, LDH level, regression measurement, imaging method and method used to determine the patient count. Potential sources of heterogeneity were explored by meta-regression with a *p* value less than 0.1. Sensitivity analysis was performed by including only high-quality non-randomized studies and low-risk RCTs and by sequentially excluding each study. Publication bias was assessed using Egger’s test and was visualized with a funnel plot. All statistical analyses were performed using the Meta and metafor packages in R (V3.6.1) [53].

## Results

### Study selection and characteristics

**Fig. 1:**
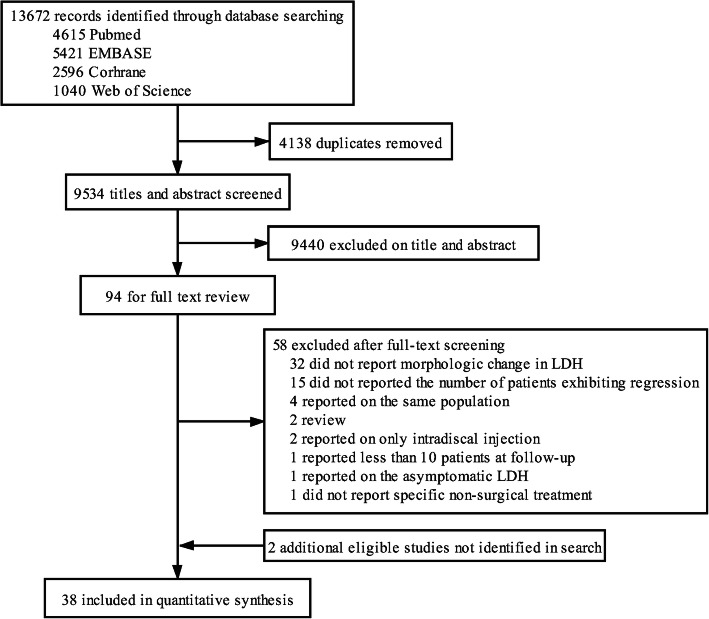
Study selection

Our initial search yielded 13,672 articles, and two were hand-selected from reference lists. A total of 38 articles were included in the final meta-analysis (Fig. 1). The non-surgical treatment used in these studies included bed rest, lumbar support, traction, spinal manipulation, physical therapy, exercise, oral steroids, analgesics, nonsteroidal anti-inflammatory agents, epidural block, caudal epidural injections, traditional Chinese medicine and alternative medicine. These articles included 5 RCTs and 33 nonrandomized studies. The studies were from Asia, Europe and North America and were from a total of 13 countries. Japan contributed seven studies; Korea and the USA each contributed five; Turkey contributed four; China, the UK and Italy each contributed three; France and Finland each contributed two; and Denmark, Germany, the Netherlands and Sweden each contributed one. The imaging examinations used were magnetic resonance imaging (MRI) in 29 studies and computed tomography (CT) in eight studies, and one study used CT at baseline and MRI at follow-up. The characteristics of the included studies are summarized in Table 1. These studies reported patient age (14–78), symptom duration (one day-ten years) and follow-up time (20 days – 6.1 years) in different formats (Table 2). A total of 16 studies did not report symptom duration, five studies did not report whether nerve symptoms were experienced by all patients or by a subset of patients, and five studies did not describe or define regression (Table 2).

**Table 1:** Characteristics of the included studies

Author	Year	Country	Study type	Quality^a^	LDH Level^b^	Measurement	Imaging method	Counting^c^	Number of patients	
									Regression	Total
El Barzouhi et al. [9]	2013	Netherlands	RCT	Low risk	single	Qualitative	MRI	A	88	95
Santilli et al. [10]	2006	Italy	RCT	Low risk	single/multiple	Qualitative	MRI	A	0	102
Fan et al. [11]	2015	China	RCT	Unclear risk	Unknow	Qualitative	MRI	A	0	158
Ahn et al. [12]	2002	Korea	Prospective	11	single	Qualitative	MRI	B	13	17
Maigne et al. [13]	1992	France	Prospective	10	single	Qualitative	CT	B	39	48
Benson et al. [14]	2010	UK	Prospective	12	single	Quantitative	MRI	B	28	32
Komori et al. [15]	1998	Japan	Retrospective	12	single	Qualitative	MRI	A	19	22
Modic et al. [16]	1995	USA	Prospective	12	single/multiple	Qualitative	MRI	B	4	16
Kamanli et al. [17]	2010	Turkey	Prospective	8	Unknown	Qualitative	MRI	A	5	26
Autio et al. [18]	2006	Finland	Prospective	12	single	Quantitative	MRI	A	51	55
Gallucci et al. [19]	1995	Italy	Prospective	10	Unknown	Qualitative	MRI	B	11	15
Ozturk et al. [20]	2006	Turkey	RCT	Low risk	single/multiple	Quantitative	CT	A	19	46
Ahn et al. [21]	2000	Korea	Prospective	12	single	Quantitative	MRI	A	25	36
Ilkko et al. [22]	1993	Finland	Unknown	7	single/multiple	Qualitative	CT	A	15	18
Delauche-Cavallier et al. [23]	1992	France	Prospective	9	single	Qualitative	CT	A	14	21
Buttermann et al. [24]	2002	USA	Prospective	10	single	Quantitative	MRI	A	52	58
Bozzao et al. [25]	1992	Italy	Prospective	10	Unknow	Qualitative	MRI	A	41	65
Broetz et al. [26]	2008	Germany	Prospective	9	Unknow	Qualitative	MRI	A	0	10
Jensen et al. [27]	2006	Denmark	Prospective	10	Unknow	Qualitative	MRI	A	65	139
Henmi et al. [28]	2002	Japan	Unknown	11	single	Quantitative	MRI	A	4	10
Takada et al. [29]	2001	Japan	Prospective	9	single	Qualitative	MRI	A	37	42
Cribb et al. [30]	2007	England	Unknown	7	single	Quantitative	MRI	A	14	15
Ellenberg et al. [31]	1993	USA	Prospective	9	single/multiple	Qualitative	CT	A	11	14
Demirel et al. [32]	2017	Turkey	RCT	Low risk	single/multiple	Quantitative	MRI	A	18	20
Hong et al. [33]	2016	Korea	Retrospective	10	single	Quantitative	MRI	A	24	28
Matsubara et al. [34]	1995	Japan	Unknown	9	single	Qualitative	MRI	B	20	32
Yukawa et al. [35]	1996	Japan	Unknown	11	single	Quantitative	MRI	A	17	30
Fagerlund et al. [36]	1990	Sweden	Prospective	12	single	Qualitative	CT	B	22	30
Kesikburun et al. [37]	2019	Turkey	Prospective	12	single	Quantitative	MRI	A	36	40
Teplick et al. [38]	1985	USA	Unknown	7	Unknown	Qualitative	CT	A	11	55
Shan et al. [39]	2014	China	Retrospective	12	single	Quantitative	MRI	A	24	30
Shin et al. [40]	2017	Korea	Prospective	8	Unknown	Qualitative	MRI	A	42	73
Komori et al. [41]	1996	Japan	Retrospective	10	single	Qualitative	MRI	A	49	77
Saal et al. [42]	1990	USA	Unknown	8	Unknown	Qualitative	CT/MRI	B	9	11
Bush et al. [43]	1992	UK	Prospective	10	single/multiple	Qualitative	CT	A	71	111
Iwabuchi et al. [44]	2010	Japan	Prospective	10	single	Qualitative	MRI	A	21	34
Yu et al. [45]	2014	China	Unknown	10	single	Qualitative	MRI	A	20	83
Lee et al. [46]	2017	Korea	Retrospective	10	single	Quantitative	MRI	A	486	505

^a^Quality of non-randomized studies was assessed following the MINORS; the risk of bias of RCTs was evaluated using a tool from the Cochrane Collaboration

^b^Some studies included only single-level SLDH patients and some studies included both single- and multiple-level SLDH patients

^c^Counting. A. The number of patients with regression was reported and was extracted from the publication. B: For studies that recorded the number of patients by the regression proportion or size interval but did not define the interval of non-regression or report the number of patients without regression, we regarded the lowest interval as the no regression range, and the number of patients outside of this interval was considered the number of patients with regression

**Table 2:** Other characteristics of the included studies

Author	Year	Age	Duration of symptom	Nerve symptom	Regression defined	Follow-up
El Barzouhi et al. [9]	2013	18–65	6–12 W	Yes	Yes	1Y
Santilli et al. [10]	2006	18–65	Less than 10 D	Yes	Yes	45D
Fan et al. [11]	2015	Unknown	Unknown	Yes	No	20D
Ahn et al. [12]	2002	19–73 (42.7)	1-10 W(median 4.5 W)	Some	Yes	3-11 M(11.9 M)
Maigne et al. [13]	1992	26–75 (45.2)	Unknown	Yes	Yes	1-48 M
Benson et al. [14]	2010	25–62 (40.4)	More than 6 W	Yes	Yes	3-42 M(13.2 M)
Komori et al. [15]	1998	20–75 (41)	2-359D(54D)	Yes	Yes	27-856D(151D)
Modic et al. [16]	1995	22–75 (49.2)	Less than 2 W	Yes	Yes	6 W-6 M
Kamanli et al. [17]	2010	(37)	Unknown	Unknown	No	4-6 W
Autio et al. [18]	2006	19–78	3–28 W	Yes	Yes	12 M
Gallucci et al. [19]	1995	27–62 (37)	Unknown	Some	Yes	6 M
Ozturk et al. [20]	2006	16–70	Less than 6 M	Some	Yes	21D
Ahn et al. [21]	2000	17–74 (39)	1–28 M	Yes	Yes	3-27 M(8.5 M)
Ilkko et al. [22]	1993	35–74 (53)	Unknown	Some	Yes	4.3–6.1Y(5.2Y)
Delauche-Cavallier et al. [23]	1992	20–64 (43)	15D-6 M(2 M)	Yes	Yes	6-27 M(12.9 M)
Buttermann et al. [24]	2002	18–70	Unknown	Yes	Yes	18 ± 10&19 ± 9 M
Bozzao et al. [25]	1992	23–65 (52)	1 M-1Y	Some	Yes	6-15 M(11 M)
Broetz et al. [26]	2008	18–65	5D-2Y(median 5 W)	Yes	No	3-7D(5D)
Jensen et al. [27]	2006	18–65 (45)	1-3 M	Yes	Yes	12 M
Henmi et al. [28]	2002	20–50	1-200D	Yes	Yes	6-12 M
Takada et al. [29]	2001	16–64 (42)	1-14 W	Yes	Yes	3-24 M(10.3 M)
Cribb et al. [30]	2007	24–73 (45)	Unknown	Yes	No	5-56 M(24 M)
Ellenberg et al. [31]	1993	28–67 (42)	Unknown	Yes	Yes	6-18 M(9.8 M)
Demirel et al. [32]	2017	50.7	Unknown	Unknown	Yes	3 M
Hong et al. [33]	2016	26–78 (50.2)	Unknown	Unknown	Yes	2-31 M(8.8 M)
Matsubara et al. [34]	1995	16–52 (36)	Unknown	Yes	Yes	3-18 M(9.7 M)
Yukawa et al. [35]	1996	14–69 (39)	Unknown	Yes	Yes	24-42 M(30 M)
Fagerlund et al. [36]	1990	14–49 (35)	6 ± 3 M	Unknown	Yes	24 M
Kesikburun et al. [37]	2019	39.7–71.5 (54.4)	4.7–7.4 M(6 M)	No	Yes	12-19 M(17 M)
Teplick et al. [38]	1985	Unknown	Unknown	Unknown	No	3 M-5Y
Shan et al. [39]	2014	20–66 (40)	2 W-6 M	Yes	Yes	6 M
Shin et al. [40]	2017	(35.8)	(2.7 M)	Yes	Yes	3Y
Komori et al. [41]	1996	18–86 (41)	0.1–8.6 M(1.8 M)	Yes	Yes	62-1208D(262D)
Saal et al. [42]	1990	Unknown	Unknown	Yes	Yes	8-77 M(25)
Bush et al. [43]	1992	17–72 (41)	1-72 M(4.2 M)	Yes	Yes	1Y
Iwabuchi et al. [44]	2010	−52	Unknown	Yes	Yes	(4.1 M)
Yu et al. [45]	2014	16–60 (38.7)	3D-10Y(16.5 M)	Yes	Yes	2-24 M
Lee et al. [46]	2017	39.08 ± 10.19	Unknown	Yes	Yes	341.38 ± 306.83D

Average values were presented in parentheses and “±”is connected to Mean and SD if available. *D* Day, *M* Month, *Y* Year

### Quality assessment

Of the 5 included RCTs, 4 showed a low risk of bias, and 1 showed an unclear risk of bias. Of the 33 included non-randomized studies, 22 were of high quality, and 11 were of moderate quality (Table 1).

### Incidence synthesis and data analysis

**Fig. 2:**
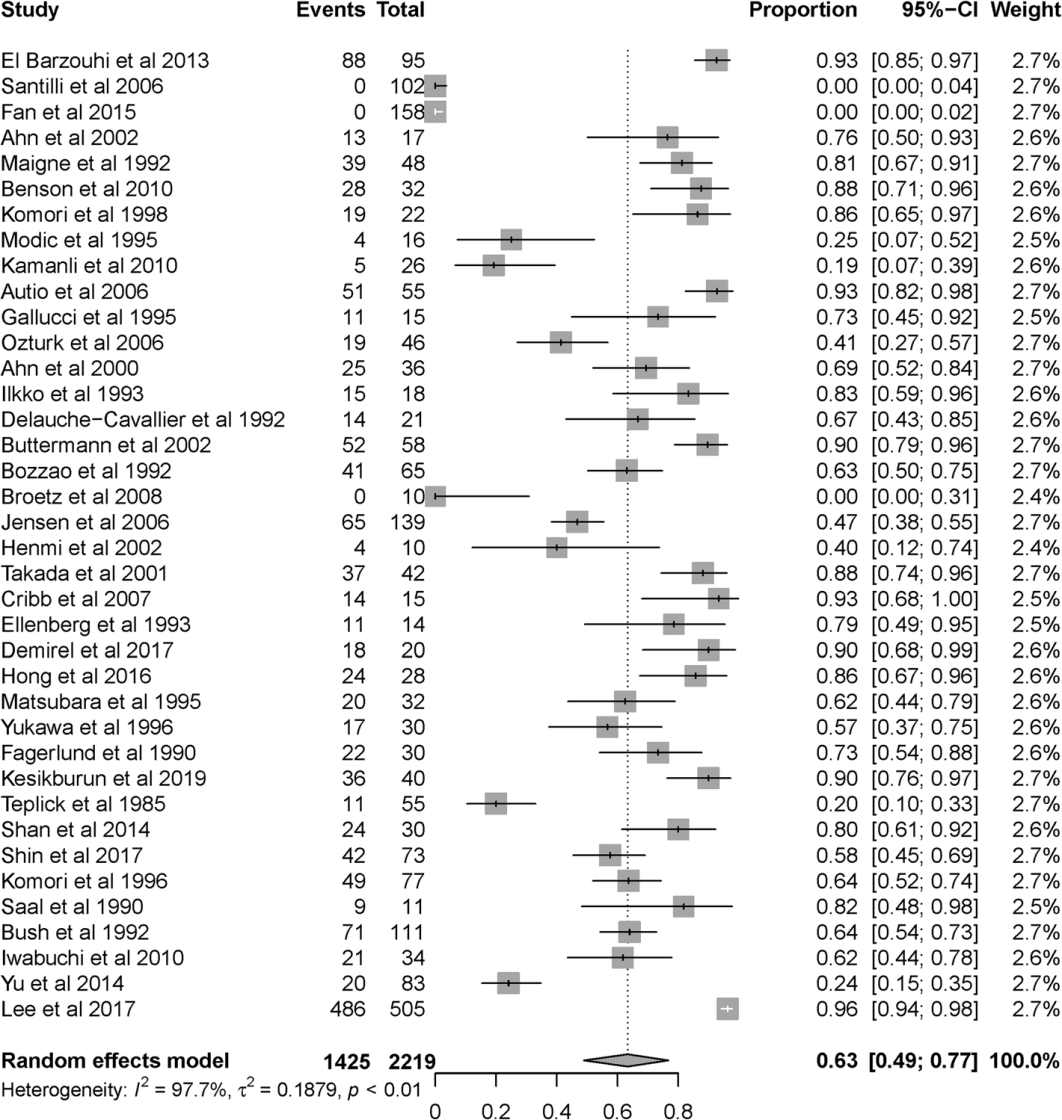
Overall IR after the non-surgical treatment of SLDH. Weights are from the random effects analysis. Grey squares represent the proportional weight of each study in the meta-analysis. The pooled incidence and CIs from studies with zero events were treated by adding 0.5 cases to both the numerator (number of patients with regression) and denominator (total number of SLDH patients)

The pooled analysis for IR after the nonsurgical treatment of SLDH included 2219 patients, 1425 of whom presented regression. The pooled IR in our study was 63% (95% CI 0.49–0.77), with significant heterogeneity among the studies (I^2^ = 97.7%, *p* < 0.001; Fig. 2).

Subgroup analyses (Table 3) showed that studies that quantitatively measured the regression of SLDH yielded statistically higher (*p* = 0.02) pooled IRs (81, 95% CI 0.69–0.91) than those that adopted qualitative methods (54, 95% CI 0.37–0.70). We repeated subgroup analyses based on the time period of the study and did not identify any secular trends in the IR of non-surgically treated patients before 2000 (65, 95% CI 0.55–0.75), from 2000 to 2009 (57%, 0.29–0.83), or from 2010 to 2019 (66%, 0.35–0.91). We found no significant regional variation within Asia (63, 95% CI 0.40–0.83), Europe (65%, 0.43–0.85), and North America (60%, 0.22–0.92). The pooled IR gradually increased in RCTs (37, 95% CI 0.00–0.88), prospective studies (67%, 0.57–0.77), and retrospective (84%, 0.65–0.97) studies. Studies of single-level SLDH patients (78, 95% CI 0.67–0.87) yielded higher pooled IRs than those that included both single- and multiple-level SLDH patients (51%, 0.17–0.86). Studies based on MRI yielded the same pooled IR (63, 95% CI 0.46–0.79) as those based on CT (63, 95% CI 0.45–0.79); the IR was calculated as 82% in Saal’s research [42], in which CT was used at baseline and MRI was used at follow-up. Studies that reported the number of patients without regression yielded lower pooled IRs (61, 95% CI 0.44–0.77) than those that did not define regression or reported the number of patients without regression (72%, 0.59–0.83).

**Table 3:** Subgroup analyses of the regression measurement, time period, region, study type, LDH level, imaging method and patient count

	Included studies (n)	Number of patients		Incidence (95% CI)	***P*** value^*^
		With Regression	Total		
**Measurement**	**38**	**1425**	**2219**	**63%(0·49–0·77)**	0.01
Qualitative	25	627	1314	54% (0·37–0·70)	
Quantitative	13	798	905	81% (0·69–0·91)	
**Time period**	**38**	**1425**	**2219**	**63%(0·49–0·77)**	0.87
Before 2000	15	353	565	65% (0·55–0·75)	
2000–2009	11	280	530	57% (0·29–0·83)	
2010–2019	12	792	1124	66% (0·35–0·91)	
**Region**	**38**	**1425**	**2219**	**63%(0·49–0·77)**	0.97
Asia	19	879	1309	63% (0·40–0·83)	
Europe	14	459	756	65% (0·43–0·85)	
North America	5	87	154	60% (0·22–0·92)	
**Study type**	**30**^a^	**1315**	**1965**	**65%(0·48–0·80)**	0.14
RCT	5	125	421	37% (0·00–0·88)	
Prospective	20	588	882	67% (0·57–0·77)	
Retrospective	5	602	662	84% (0·65–0·97)	
**LDH level**	**29**^b^	**1241**	**1667**	**72% (0·58–0·84)**	0.17
Single	22	1103	1340	78% (0·67–0·87)	
Single/multiple	7	138	327	51% (0·17–0·86)	
**Imaging method**	**37**^c^	**1416**	**2208**	**63%(0·48–0·76)**	0.97
CT	8	202	343	63% (0·46–0·79)	
MRI	29	1214	1865	63% (0·45–0·79)	
**Counting**^d^	**38**	**1425**	**2219**	**63%(0·49–0·77)**	0.33
A	30	1279	2018	61% (0·44–0·77)	
B	8	146	201	72% (0·59–0·83)	

*P value is from the test for subgroup differences (random effects model)

^a^Eight studies did not report study type

^b^Nine studies did not report LDH level

^c^One study used CT at basline and MRI at follow-up

^d^Counting. A. The number of patients with regression was reported and was extracted from the publication. B. For studies that recorded the number of patients by the regression proportion or size interval but did not define the interval of non-regression or report the number of patients without regression, we regarded the lowest interval as the no regression range, and the number of patients outside of this interval was considered the number of patients with regression

**Fig. 3:**
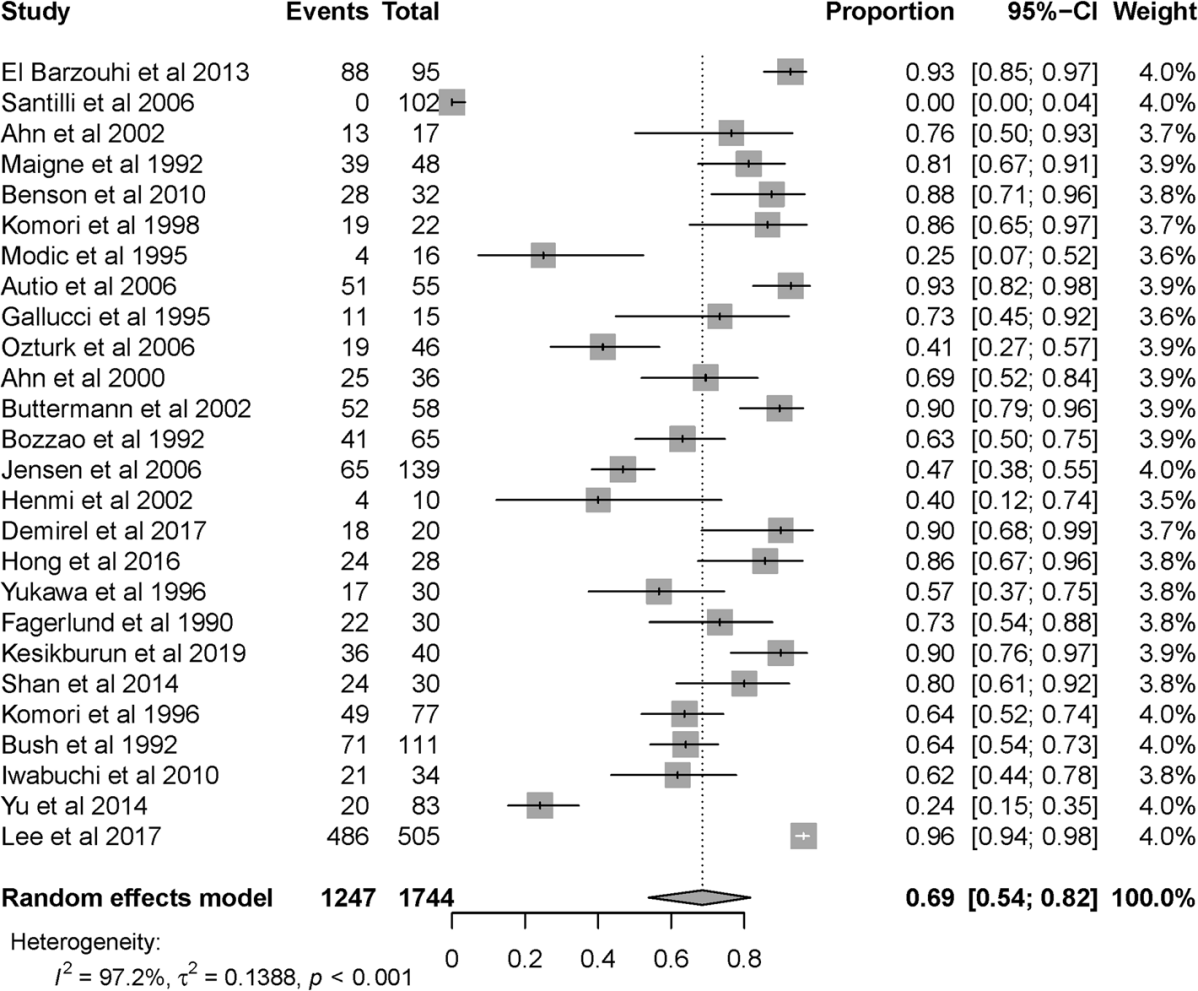
Forest plot for the meta-analysis of high-quality nonrandomized studies and low-risk RCTs

Meta-regression showed that study types (R^2^ = 41.94% *p* = 0.02), LDH levels (R^2^ = 31.53%, *p* = 0.05), and regression measurements (R^2^ = 41.94% *p* = 0.02) contributed to the heterogeneity. There was no significant change in the pooled IR (69, 95% CI 0.54–0.82) or heterogeneity (I^2^ = 97.2%, *p* < 0.001) when only high-quality non-randomized studies and low-risk RCTs were included (Fig. 3). The pooled IR varied from 62 to 66% after the sequential omission of any single study.

### Publication bias

**Fig. 4:**
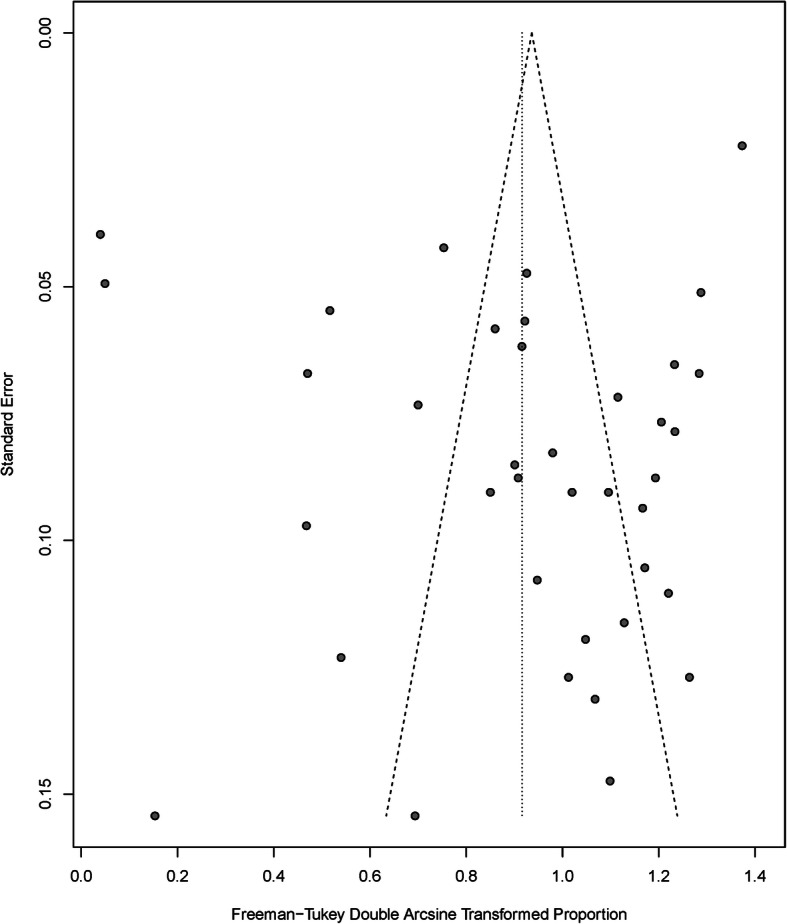
Funnel plot of incidence

Egger’s test suggested that there was no publication bias (*p* = 0.46). No asymmetric patterns were seen in the funnel plot (Fig. 4).

## Discussion

We found an IR of 63% after the non-surgical treatment of SLDH in the present systematic review and meta-analysis, with significant heterogeneity among the studies. Our pooled IR needs to be interpreted with caution.

We comprehensively searched for studies that potentially reported morphological changes in SLDH during clinical follow-up and that investigated the regression of SLDH. We conducted a wide database search, and a small number of articles were included. Because follow-up is a necessary step to study regression, “follow up” or “follow-up” or “outcome” or “result” was included in the search terms. The use of these search terms resulted in the retrieval of a large number of articles. However, there are so many non-surgical treatment methods for SLDH in the world that it is impossible to limit the specific non-surgical treatment methods in the literature search process. In addition, studies that compared the results of surgical and non-surgical treatment may have reported the morphological changes in herniated discs of the non-surgically treated patients, making it impossible to exclude studies on surgery. As a result, 13,672 articles were identified, more than half of which were studies on surgery for SLDH, and a small number of articles were included. Both RCTs and non-randomized studies were included in our study. The pooled IR in our study was similar to the IR of 66.66% that was reported in a previous review of 11 studies [54], and these IR values can be considered quantitative data that can inform clinical decisions regarding SLDH.

The highest IR (96%) was documented by Lee with an average follow-up of 341 days [46], suggesting that we should seriously consider the probability of SLDH regression. Three studies reported no regression with follow-ups of 45 days [10], 20 days [11], and a median of 5 days (3–7 days) [26], suggesting that SLDH regression should not be expected to occur within one and a half months of symptom onset. The average of the IRs reported in the included studies was 63%, which is the same as the pooled IR of the meta-analysis, and 7 studies reported IRs of approximately 63%: Ahn [21] reported an IR of 69% with an average follow-up time of 8.5 months, Delauche-Cavallier [23] reported an IR of 67% with an average follow-up time of 12.5 months, Bozzao [25] reported an IR of 63% with an average follow-up time of 11 months, Matsubara [34] reported an IR of 62% with an average follow-up time of 9.7 months, Komori [41] reported an IR of 64% with an average follow-up time of 262 days, Bush [43] reported an IR of 64% with an average follow-up time of 1 year, and Iwabuchi [44] reported an IR of 62% with an average follow-up time of 4.1 months. According to Iwabuchi’s report, which reported an IR that was consistent with the average of the IRs reported for the included studies and had a follow-up time of 4.1 months [44], we suggest that 4 months after onset is an important time point for imaging. The follow-up time of the other 6 studies with IRs of approximately 63% ranged from 8.5 to 12.9 months, with an average of 10.5 months. Therefore, we suggest that 10.5 months after onset is another important time point for imaging. There were 4 studies that reported long-term follow-up, with an average duration of more than 24 months: Fagerlund [36] reported an IR of 73% with a follow-up of 24 months, Yukawa [35] reported an IR of 57% with an average follow-up of 30 months, Shin [40] reported an IR of 58% with a follow-up of 3 years, and Ilkko [22] reported an IR of 83% with an average follow-up of 5.2 years. The IR trend reported by these articles over time was inconsistent; some reported that IR increased over time to above the average IR, some reported that IR decreased over time to fall below the average IR, and no secular trends were identified for long-term follow-up.

We did not classify SLDH during the data synthesis, as most of the studies included in our meta-analysis did not include classifications; this is in contrast to another review that calculated IR based on 9 articles reporting that sequestration, extrusion, protrusion and bulging were present in 96, 70, 41 and 13% of patients, respectively [55]. These IR classifications provide a more detailed reference. The probability of SLDH regression should be considered in clinical practice according to the guidelines of the North American Spine Society [48], and we provided an extensive summary of estimated IRs as evidence. Together with existing evidence, our research shows that the regression of SLDH should be fully considered by clinical decision makers. For patients without absolute indications for surgery, the regression of SLDH can be considered very likely, and surgery may be avoided for most patients. As some SLDH patients who were treated non-surgically did not experience regression, the effective prediction of SLDH regression should be explored in the future.

Our study revealed that the study types, LDH levels and regression measurements contributed to the heterogeneity. The increase in the risk of selection bias in the three study types (RCTs, prospective studies and retrospective studies [56, 57]) was consistent with the increase in the pooled IR of the three types of studies, explaining the heterogeneity observed among different study types. The pooled IR of studies that included only single-level SLDH patients was higher than that of those including both single- and multiple-level SLDH patients. Because there are usually more herniated disc tissues in single-level SLDH than in multiple-level SLDH, which induces a more robust inflammatory response, and the most likely mechanism underlying regression is an inflammatory response directed against the herniated disc tissues [58, 59], patients with single-level SLDH are more likely to experience regression than patients with multiple-level SLDH. We also found that studies that included quantitative measurements tended to report higher IRs for SLDH than studies that qualitatively measured LDH. The quantitative methods used in these included studies included 3D volume measurements and cross-sectional area measurements, while the qualitative methods used were visual estimations. Quantitative measurements were performed in millimetres or centimetres, and in some studies, they were accurate to one decimal place. In general, quantitative measurements are better for detecting small dimensional changes than visual assessments. In addition, intervertebral discs are three-dimensional irregularly shaped tissues, making it difficult to capture small changes in their volume on planar images using visual estimation. Quantitative measurements made it easier to record slight changes in the size of the discs on sagittal and cross-sectional views or changes in volume that are rarely detected by visual inspection due to the occurrence of slight changes in multiple directions. Both imaging methods have obvious defects that may cause inaccuracies. For quantitative measurements, it was impossible for the follow-up images to use the exact same slices that were initially scanned [60, 61]. For qualitative measurements, unclear borders and the three-dimensional characteristics of LDH made the judgement of regression inaccurate, especially for visual estimations. In the future, a more standardized and reliable method for determining the occurrence of SLDH regression needs to be established. Other factors, such as age, symptom duration, type of non-surgical treatment and follow-up time, may play a role in heterogeneity, these factors were documented in the included studies, but sufficient information was not available for determining whether these factors contributed to the heterogeneity.

Our study has limitations. We included studies in the meta-analysis without limiting the criteria or measurements for regression to ensure the robustness of IR synthesis, which inevitably led to the inclusion of sources of heterogeneity. Presently, there is no clear definition of the time frame for SLDH regression. The follow-up period of some of the included studies may not have been appropriate or long enough to observe the presence of SLDH regression, making the reported IR lower than the actual IR.

## Conclusions

Our meta-analysis results supplement the guidelines of the North American Spine Society on the IR [48]. We revealed an overall IR of 63% among patients with SLDH who were treated non-surgically, thus providing clinical decision makers with quantitative evidence of IR. The probability of regression after the non-surgical treatment of SLDH should be fully considered before making decisions regarding surgery. Based on our systematic review, we suggest a follow-up timeline that consists of the time points 4 and 10.5 months after onset when deciding whether to perform surgery for SLDH. Surgery can be considered for patients with severe symptoms who do not experience regression after 4 months of onset, and we highly recommend surgery for those who do not experience regression after 10.5 months of onset.

## Supplementary information

**Additional file 1.**

## Data Availability

Not applicable.

## Abbreviations

SLDH: Symptomatic lumbar disc herniation
IR: Incidence of regression
LDH: Lumbar disc herniation
RCTs: Randomized controlled trials
MINORS: Methodological Index for Non-Randomized Studies
PRISMA: Preferred Reporting Items for Systematic Reviews and Meta-Analyses
CIs: Confidence intervals
