# Supplementary material for: The incidence of regression after the non-surgical treatment of symptomatic lumbar disc herniation: a systematic review and meta-analysis
Source: BMC Musculoskelet Disord. 2020 Aug 10;21:530. doi: 10.1186/s12891-020-03548-z (PMC7419225; doi:10.1186/s12891-020-03548-z)
Supplement: Supplementary file 1 — Additional file 1. [file 12891_2020_3548_MOESM1_ESM.pdf]

Additional file. Search strategy.

Search	Query
#1	Search ((Disc[Title]) OR "nucleus pulposus"[Title]) OR "nucleus pulpous"[Title]
#2	Search ((((extru*[Title]) OR Slipped[Title]) OR Hernia*[Title]) OR Displace*[Title]) OR Prolaps*[Title]
#3	#1 AND #2
#4	Search (Intervertebral Disc Displacement[MeSH Terms]) OR Intervertebral Disc Degeneration[MeSH Terms]
#5	#3 OR #4
#6	Search ((((((((((((((((((Outcome?[Title]) OR Result?[Title]) OR "Follow up"[Title]) OR Follow-up[Title]) OR observ*[Title]) OR Regress*[Title]) OR resorpt*[Title]) OR absorption?[Title]) OR absorb*[Title]) OR reduc*[Title]) OR decreas*[Title]) OR resolution?[Title]) OR change*[Title]) OR versus[Title]) OR Compar*[Title]) OR “natural history”[Title]) OR Course*[Title]) OR imag*[Title]) OR MRI[Title]) OR “Computed Tomography”[Title]) OR CT[Title]
#7	#5 AND #6

PubMed search performed on September 16, 2019.
